# Service user perspectives on social prescribing services for mental health in the UK: a systematic review

**DOI:** 10.1177/17579139231170786

**Published:** 2023-05-26

**Authors:** M Cooper, D Flynn, L Avery, K Ashley, C Jordan, L Errington, J Scott

**Affiliations:** School of Health and Life Sciences, Teesside University, Tees Valley TS1 3BX, UK; Faculty of Health and Life Sciences, Northumbria University, Newcastle upon Tyne, UK; School of Health and Life Sciences, Teesside University, Tees Valley, UK; School of Health and Life Sciences, Teesside University, Tees Valley, UK; School of Health and Life Sciences, Teesside University, Tees Valley, UK; School of Biomedical, Nutritional, and Sport Sciences, Newcastle University, Newcastle upon Tyne, UK; Faculty of Health and Life Sciences, Northumbria University, Newcastle upon Tyne, UK

**Keywords:** systematic review, social prescribing, qualitative synthesis, mental health, public health, primary care

## Abstract

**Aim:**

To thematically synthesise adult service users’ perspectives on how UK-based social prescribing services support them with their mental health management.

**Methods:**

Nine databases were systematically searched up to March 2022. Eligible studies were qualitative or mixed methods studies involving participants aged ⩾ 18 years accessing social prescribing services primarily for mental health reasons. Thematic synthesis was applied to qualitative data to create descriptive and analytical themes.

**Results:**

51,965 articles were identified from electronic searches. Six studies were included in the review (*n* = 220 participants) with good methodological quality. Five studies utilised a link worker referral model, and one study a direct referral model. Modal reasons for referral were social isolation and/or loneliness (*n* = 4 studies). Two analytical themes were formulated from seven descriptive themes: (1) person-centred care was key to delivery and (2) creating an environment for personal change and development.

**Conclusions:**

This review provides a synthesis of the qualitative evidence on service users’ experiences of accessing and using social prescribing services to support their mental health management. Adherence to principles of person-centred care and addressing the holistic needs of service users (including devoting attention to the quality of the therapeutic environment) are important for design and delivery of social prescribing services. This will optimise service user satisfaction and other outcomes that matter to them.

## Introduction

Social prescribing in the UK is defined by the Social Prescribing Network as ‘a means of enabling professionals to refer people to non-clinical services to support their health and wellbeing’.^1^ However, multiple definitions of social prescribing are used in research. It has been proposed that definitions in the UK are influenced by current politics, health status, care use, and capacity,^2^ which potentially leads to an oversimplification of social prescribing and its capability to influence public health outcomes.^2^ Social prescribing is typically delivered in primary care or community settings; however, research is currently expanding its application to other areas of healthcare such as secondary care^3,4^ and pre-hospital care.^5^ Social prescribing addresses many facets of public health, such as social isolation and loneliness,^6–8^ weight management,^9^ and mental health and wellbeing in the wider population.^10^

Central to the social prescribing pathway is a link worker, a role with many title iterations such as community links practitioner, social navigator, or community care coach. Link workers are defined by National Health Service (NHS) England to ‘connect people to community-based support, including activities and services that meet practical, social, and emotional needs that affect their health and wellbeing’.^11^ Link workers have a person-centred and needs led conversation with service users to identify possible areas of support needed. The link worker will then offer a referral to the type of support required. A service user may see a link worker multiple times over a set period and is based on the link worker’s professional judgement.

The consensus across multiple systematic reviews is there is significant promise for social prescribing services to create meaningful changes in public health. However, research is yet to provide a sufficient evidence base to permit conclusions about effectiveness of social prescribing for health outcomes and healthcare service utilisation.^12,13^ Previous reviews of social prescribing have tended to focus on methodology, delivery, or referral pathways,^12–14^ but have lacked a specific focus on an exploration of the evidence for populations with specific needs, such as people living with mental health conditions. A recent review of social prescribing services targeting mental health and wellbeing outcomes^15^ identified a range of active ingredients utilised by interventions (intensity, underpinning theory, and theory-linked behaviour change techniques) but was unable to establish effectiveness due to issues with methodological quality.

Mental health is core to the NHS Long Term Plan,^16^ with the number of people in contact with mental health services in England reaching 1.62 million at the end of May 2022.^17^ The prevalence of people in the UK requiring support for mental health is also increasing, with estimates of > 50% increase from 2017 to 2019 to April 2020, which was the period following national lockdowns in response to the COVID-19 pandemic.^18^ The most common mental health conditions requiring support are anxiety and depression,^19^ with an estimated 15% of people at any one time in the UK living with a mental health condition.^20^ As part of the NHS Long Term Plan,^16^ there is a drive towards personalised care.^11^ One of the core personalised care services is social prescribing, which is underpinned by significant investment at the national level in England and is part of the six pillars of the personalised healthcare agenda.^16^

Research studies have reported that social prescribing can impact positively on mental wellbeing, self-confidence, self-esteem, and social isolation.^12,21,22^ Individuals engaging in social prescribing services report greater independence and purpose,^10^ increased self-confidence,^10,23^ and increased numbers of social engagements.^24^ These findings have been attributed to trusting relationships formed with link workers and the supportive environment created by services that receive referrals for social prescriptions,^10,21–25^ which enables the creation of a safe space for individuals to explore their current issues and build the skills to self-manage their health.^24,26^

Social prescribing research has often used qualitative methods and the application of theory, such as Self-determination Theory^24^ and Social Identity Theory,^27^ to develop a more robust evidence base on how and why social prescribing works. However, there is no universally agreed theoretical underpinning for social prescribing.^15^ One of social prescribing’s key features is the ability to be highly personalised and tailored to individual needs. Where studies have looked at specific social prescribing services for people with mental health needs, they have concluded (based on quantitative outcomes) a personalised care approach to the delivery of services provided an effective means of reducing mental distress and improving mental health and wellbeing outcomes.^22,28,29^ However, systematic review evidence has identified few social prescribing services report on explicit criteria for person-centredness.^15^

To elucidate the theory and associated mechanisms underpinning effective social prescriptions for people living with mental health conditions in the UK, a systematic synthesis of the qualitative literature with a specific focus on service users’ experiences is warranted. Therefore, this systematic review aimed to synthesise qualitative evidence generated from adults with lived experience of mental health conditions who have used social prescribing services in the UK to manage their mental health.

## Methods

### Design

This systematic review was conducted in accordance with the Preferred Reporting Items for Systematic Review and Meta-Analysis (PRISMA) guidelines^30^ Previously we reported on a narrative synthesis of quantitative outcomes from UK-based studies of social prescribing in the context of mental health,^15^ which adhered to a review protocol registered with PROSPERO (CRD42020167887).^31^ Using the same search and adhering to the review protocol, this qualitative systematic review synthesises evidence from service users in the UK who have accessed and received social prescriptions for their mental health. A completed PRISMA checklist is provided in supplementary file 1.

### Search strategy

Nine electronic databases were searched from inception to 21 March 2022: Cochrane Databases of Systematic Reviews, The Cochrane Central Register of Controlled Trials, CINAHL, Cochrane Protocols, Embase, Medline, PsycInfo, Scopus, and Web of Science. Scoping searches were undertaken to identify search terms relevant to social prescribing and mental health. The search strategy was subsequently developed and conducted by an information scientist (LE). Searches were restricted to UK-based studies (to ensure relevancy and transferability of findings to UK healthcare systems) published in the English language. Hand and citation searching of included studies were conducted using Google Scholar. The search strategy applied to all electronic databases is available in supplementary file 2.

### Inclusion and exclusion criteria

Included studies were social prescribing services (and/ or interventions depending on terminology used) based in the UK involving adults aged ⩾18 years referred for a social prescription for mild to moderate mental health reasons (including but not exclusive to a diagnosis and/or experiencing symptoms of anxiety, depression, social isolation, loneliness). Studies were qualitative study designs (interviews or focus groups) or mixed methods, where service user data could be extracted independently from all data reported. Studies were excluded if there was no referral or signposting to either a link worker or group/ service and/or did not report any qualitative data.

### Screening

All results from the search were uploaded to EndNote X9 and deduplicated. Titles and abstracts were screened by one reviewer (MC) and 20% screened independently by a second reviewer (CJ). The full text of all studies retained after title and abstract screening were reassessed by three reviewers independently (MC, DF, JS) using a study selection form. Any disagreements at both stages of study selection that could not be resolved were discussed with a fourth reviewer (LA) who made the final decision about inclusion.

### Data extraction

A structured data extraction form was developed to capture relevant information on study characteristics (country of origin, aims, design, data collection and analysis methods, inclusion/exclusion criteria, sampling method, sample size), model of social prescribing, timing of data collection (currently engaging with a social prescribing service, or post engagement with a social prescribing service), methodological quality, and qualitative outcome data. The data extraction form was piloted by two reviewers (MC, CJ) using three included studies. Data were subsequently extracted from all included studies by one reviewer (MC) and verified by a second reviewer (KA). Any discrepancies in data extraction were resolved by discussion.

Methodological quality assessment was ascertained using the Critical Appraisal Skills Programme Qualitative Study Design Checklist^32^ applied to all included studies by two reviewers working independently (MC, JS). Studies were deemed to be either ‘very valuable’ (>15 points), ‘valuable’ (between 10 and 15 points), or ‘not valuable’ (<10 points) to the overall contribution of knowledge based on the overall score assigned (max score = 20 points).

### Data synthesis

Thematic synthesis was used to analyse qualitative data and involved three stages of analysis: stage 1 line-by-line coding of the findings, stage 2 development of the descriptive themes, and stage 3 generation of analytical themes.^33^ All descriptive text and quotes within the sections of studies labelled ‘results’ or ‘findings’ were eligible for coding.^33^

#### Stage 1: line -by- line coding

Included studies were coded line-by-line by one reviewer (MC) for meaning and content. Direct quotes presented in the results section of individual papers were not included in the coding of this review because they provided insufficient representation of the themes. However, direct quotes were used to provide further evidence and context to the themes generated in stage 3. This is consistent with previous thematic syntheses in health research.^34,35^ To ensure the translation of concepts between studies, without losing relevance and context, only service user data (based on the aims of the research) were coded.^33^ Stage 1 generated a ‘bank’ of ‘free’ codes.

#### Stage 2: organisation of ‘free codes’ into related areas to construct themes

All codes in stage 1 were organised into higher order themes by MC and discussed with three reviewers (JS, LA, DF) to establish consistency. Titles or labels reported within text of studies were not considered at this stage. The content and descriptions of themes reported directed theme generation. The stage 2 process was iterative and occurred multiple times to ensure consistency with organisation.

#### Stage 3: generating analytical themes

Stage 3 of synthesis of results from the individual studies was used to generate new analytical and associated descriptive (sub)-themes. MC and JS generated new analytical themes, which were discussed with LA and DF to produce a consensus on final themes. The final themes are then presented in tabular format and a thematic tree. Supporting quotes from individual papers were included in the table to provide credibility and additional context to the final themes.

## Findings

**Figure 1:**
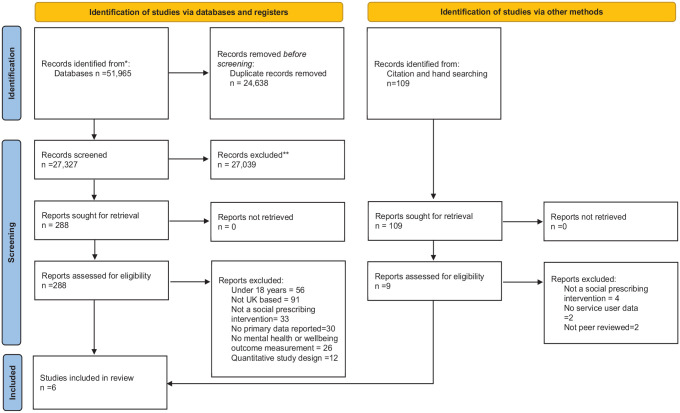
PRISMA diagram. PRISMA: Preferred Reporting Items for Systematic Review and Meta-Analysis.

A total of 51,965 studies were identified from the electronic searches with an additional 109 identified through hand and citation searching (Figure 1). Full-text papers (*n* = 288) were assessed for eligibility, with six papers fulfilling all review criteria.^7,21–23,28,36^

### Study characteristics

A summary of the six included study characteristics is provided in Table 1. The combined sample size across the six studies was 220 participants. Four studies were conducted in England,^7,21–23^ one in Scotland,^36^ and one in Wales.^28^ All studies used semi-structured interviews and thematic analysis to analyse qualitative data.^7,21–23,28,36^ All six offered social prescriptions to activities or services in the voluntary, community and social enterprise sector.^7,21–23,28,36^ Models of social prescribing were categorised according to Husk *et al*.^37^ Five studies used a link worker referral model involving an initial referral by either a general practitioner (GP), practice nurse, healthcare assistant, or charity to a link worker.^7,21,22,28,36^ One study used a model that directly referred (referral made from a mental health professional based in primary or secondary care, directly to the community organisation that was delivering the social prescribing service) people to an activity/service.^23^ Three studies collected data from service users after engagement with social prescribing services.^7,23,36^ One study collected data when service users were currently engaged with a service.^21^ Two studies collected data during and after engagement with social prescribing services.^22,28^

**Table 1:** Summary of study characteristics.

Author[s]	Country	Sample size	Age years (mean)	Sex (% of sample)	Reported participant ethnicity^a^	Reported employment status	Reason for referral	Model of social prescribing based on Husk *et al*^37^ (timing of data collected from service users)	Methodological Quality Assessment Score (Max 20)
Stickley and Hui^23^	England	*N* = 16	No data	50% Female 50% Male	White-British (*n* = 13), Black-British (*n* = 1), Asian (*n* = 1), Afro-Caribbean (*n* = 1)	Not reported	Mental health needs^b^	Direct referral model^c^ Data collected from service users after engagement with social prescribing services, arts-based activities	19 (Very valuable)
Moffatt *et al*.^21^	England	*N* = 30	62.0	47% Female 53% Male	White-British (*n* = 24), Black Minority Ethnic (*n* = 5), White Irish (*n* = 1)	Employed (*n* = 4) Retired (*n* = 14) Unemployed (*n* = 12)	Social isolation and loneliness	Link worker model Data collected from service users during engagement with social prescribing services, various activities	18 (Very valuable)
Kellezi *et al*.^7^	England	*N* = 19	60.4	63% Female 32% Male	White and/or British (*n* = 16)^d^	Employed (*n* = 9) Retired (*n* = 10)	Loneliness	Link worker model Data collected from service users after engagement with social prescribing services, various activities	17 (Very valuable)
Wildman *et al*.^22^	England	*N* = 24	No data	46% Female 54% Male	Not reported	Employed (*n* = 3) Retired (*n* = 10) Unemployed (*n* = 11)	Social isolation	Link worker model Data collected from service users during and after engagement with social prescribing services, various activities	18 (Very valuable)
Roberts and Windle^28^	Wales	*N* = 120	76.7	82% Female 18% Male	Not reported	Not reported	Anxiety depression stress	Link worker model Data collected from service users during and after engagement with social prescribing services, various activities	15 (Valuable)
Hanlon *et al*.^36^	Scotland	*N* = 12	46.5^e^	50% Female 50% Male	Not reported	Not reported	Psychological /social problems^f^	Link worker model Data collected from service users after engagement with social prescribing services** various activities	20 (Very valuable)

SPS: Social Prescribing Services.

a Terminology used by study authors.

b Mental Health Needs – any of the following: social isolation, loneliness, anxiety, or depression.

c Direct Referral Model – Referral made from a mental health professional based in primary or secondary care, directly to the community organisation that delivered the social prescribing intervention.

d Data collection period was not specified but was inferred based on description within the study.

e Calculated by study authors (MC and CJ).

f No further detail was provided.

The most common reasons for referral were social isolation and/or loneliness (*n* = 4).^7,21–23^ Other reasons were anxiety, depression, psychological/ social problems, and mental health needs.^7,21–23,36^ Mean age of participants across studies ranged from 47^36^ to 77^28^ years. Age data were not reported by two studies.^22,23^ Two studies reported an even distribution between male and female participants,^23,36^ two studies reported more female participants,^7,28^ and two studies reported more male participants.^21,22^ The ethnicity of participants was reported in three of out the six studies, using non-UK census categories.^7,21,23^ Across these three studies, 54 participants were reported as British and/or White (White and/or British, White-British, Black-British), five participants as Black Minority Ethnic, one participant as White-Irish, and one participant as Asian. Employment status was reported by three out of six studies.^7,21,22^ Across these three studies, 16 were employed, 34 had retired, and 23 were unemployed.

### Methodological quality assessment

Methodological quality assessment for each included study can be found in supplementary file 3. The overall score (maximum 20 points) allocated to each of the studies can also be seen in Table 1. Overall studies scores ranged from 15^28^ to 20.^36^ All six studies provided a clear statement of aims and employed appropriate research designs and associated methodologies. All studies used appropriate recruitment and data collection strategies that were consistent with the research aims.^7,21–23,28,36^ One study clearly and adequately considered the relationship between participants and researchers.^36^ Four studies explicitly reported an ethical statement.^21–23,36^ Five studies provided explicit details of a sufficiently rigorous method of data analysis.^7,21–23,36^ All six studies provided a clear statement of findings.^7,21–23,28,36^ and their contribution to knowledge, including the transferability of the conclusions.^7,21–23,28,36^ Five studies reported a new area of further research or understanding of social prescribing.^7,21–23,36^

Overall, five out of the six studies were deemed to be ‘very valuable’^7,21–23,36^ to the field and one as ‘valuable’.^28^

### Findings of thematic synthesis

**Figure 2:**
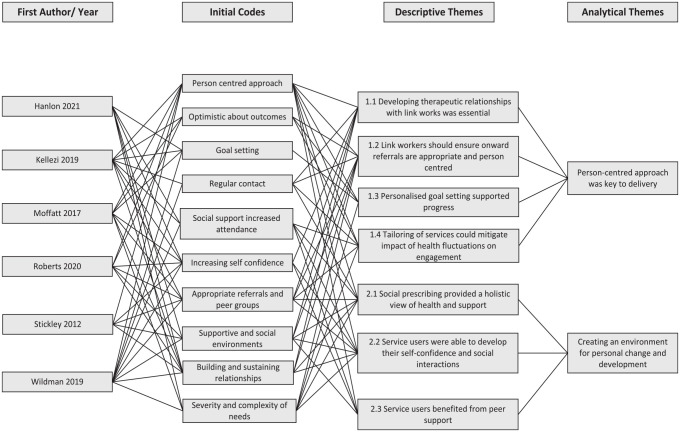
Thematic tree diagram

Two main analytical themes were developed: (1) person-centred care as key to delivery and (2) creating an environment for personal change and development. These two themes were generated by organising 10 codes into seven descriptive themes. A hierarchical thematic tree structure (figure 2) provides an overview of theme generation, including how each stage of the synthesis can be mapped onto the original studies. Supplementary file 4 provides additional context to the thematic tree structure by providing a summary of the analytical and descriptive themes. Exemplar codes (taken from the descriptions of themes reported) and direct quotes (quotes reported within individual studies results) to provide context and credibility (where available).

#### Person-centred approach was key to delivery

Across all six included studies, there was consistent reporting of a person-centred approach being preferred and valued by service users.^7,21–23,28,36^ This was reported across several aspects of the social prescribing service, including goal setting, flexible support and tailored referrals based on individual preferences and is represented in all four of the associated descriptive themes. Data indicate the link worker is central to ensuring a person-centred care approach and providing the required level and type of support to service users and aid management of their mental health:

A central part of the Link Worker role was to facilitate engagement with other services, The level and type of support offered to facilitate engagement varied and was balanced against service users’ need and readiness to engage with other services.^21^

Within the analytic theme of person-centred care, the four descriptive themes identified from the data were: (1.1) developing therapeutic relationships with link workers was essential; (1.2) link workers should ensure onward referrals are appropriate and person-centred; (1.3) personalised goal setting support progress; and (1.4) tailoring of services could mitigate impact of health fluctuations on engagement.

##### Developing therapeutic relationships with link workers was essential

The quality of the relationship between the service user and the link worker was considered essential in six of the included studies.^7,21–23,28,36^ Better quality relationships were characterised by a person-centred care approach, which aided the development of a therapeutic alliance. Service users reported ‘feeling at ease and relaxed’^21^ and ‘well-matched’^28^ with their link worker, which allowed for more open conversations about what support they needed for their mental health. Studies reported two factors driving quality relationships, trust and openness, when reporting on service users’ views about the relationship with their link worker. Having both trust and openness enabled service users to settle into socially prescribed activities and benefit from support that is tailored to their mental health needs.

##### Link workers should ensure onward referrals are appropriate, and person-centred

Appropriateness of onward referrals by link workers to support and activity services, in terms of the service users’ practical and health needs, was a prominent theme across five studies. Where service users felt they were referred to a service for activities that did not meet their needs or preferences, naturally they did ‘not feel positive about the social prescribing pathway’.^7^ However, when an onward referral was based on their mental health needs and preferences (within a person-centred care approach), service users reported them as ‘extremely helpful, particularly the combination of expert and peer-led advice on coping and symptom management strategies’.^21^ Themes within studies strongly suggested that service user engagement hinged on whether referrals met their mental health needs or not, as this directly influenced the way they would interact with services.^36^ Often referrals to peer support groups were reported as adding to the effectiveness of social prescribing, helping service users to build meaningful relationships in the future, ‘often formed through group activities which had been suggested or organised’^36^ by link workers.

##### Personalised goal setting supported progress

Themes reported across four of the six studies^7,21,22,36^ reflected on how service users benefitted from having ‘realistic, progressive and personalised goal-setting’.^21^ Service users would subsequently be more motivated to achieve their mental health goals, if there they felt they were attainable and allowed for more gradual progress over time. These four studies described how the link worker was key to working with clients in a collaborative way ensuring goals were person-centred. Themes generated from the individual studies discussed a collaborative approach where service users could ‘voice their priorities and have control over what goals were set’^36^. Having a goal in place supported service users’ mental health and progress towards meeting their priorities.

##### Tailoring of services could mitigate the impact of health fluctuations on engagement

The fluctuations in mental health conditions service users experienced impacted negatively on their motivation to engage with social prescribing services, Two studies^21,22^ reported this as a challenge but accepted it was something social prescribing services could work with rather than against. As well as fluctuations in mental health being acknowledged, it was evident service users also experienced ‘unanticipated health shocks or trauma . . . [or] psychological burden of living with (long term conditions)’^22^ that also impacted negatively on engagement. Tailoring services so service users were supported through these periods mitigated to some extent their concerns ‘about not always being able to attend’,^21^ and this flexibility helped to support their continued (re-)engagement.

#### Creating an environment for personal change and development

A second analytical theme encompassed how social prescribing can create the opportunity for individuals to develop their skills to manage their mental health and self-confidence to improve all aspects of their mental health. Within this analytical theme, there were three descriptive themes: (2.1) social prescribing provided a holistic view of health and support; (2.2) service users were able to develop their self-confidence and quality of social interactions; and (2.3) service users benefitted from peer support.

##### Social Prescribing provided a holistic view of health and support

Five studies^7,21–23,28^ reported that service users ‘believed that (social prescribing) was qualitatively different from their experiences with other health (services)’.^7^ Service users reported that they received support for anything that was affecting their health, whereas their previous experiences with health professionals involved focusing on one aspect of their health (e.g. just physical health). This holistic approach taken by social prescribing and link workers was considered more appropriate for their needs than ‘what was available or possible through the GP’.^21^ Service users had more time to discuss their mental health needs with link workers and felt better understood, which ‘brought hope and meaning to life’.^23^ Not only did the holistic approach to dealing with complex mental health needs appear to impact positively on health outcomes, service users’ also ‘said they were more confident, happier, and feeling better with an improved outlook on life’.^28^

##### Service users were able to develop their self-confidence and social interactions

Increasing service users’ self-confidence across many aspects of their lives, primarily around mental health and social interactions was reported across all six studies.^7,21–23,28,36^ Included studies reported themes suggesting that service users’ self-confidence increased following engagement with a social prescribing service and link workers. Increased self-confidence was associated with link workers ‘building self-confidence, self-reliance and independence. . .managed through ongoing support and persistence in finding the right motivational tools for the individual’.^21^ Link workers supported service users to ‘re-build and re-establish themselves’^23^ by improving their self-confidence and equipping them with the skills to feel more in control of their lives and care, including more and better-quality social interactions. By improving self-confidence and social interactions, studies generated themes suggesting that service users’ mental health improved from engaging with link workers.^7,21–23,28,36^

##### Service users benefitted from peer support

Across all six of the included studies, authors highlighted the impact that peer support had on service users health and management of their needs.^7,21–23,28,36^ Social prescribing offered the support pathway to allow service users to build their social networks and ‘increase social contact and the change to make friends with people in a similar situation’.^22^ Interacting socially with others gave service users a feeling of acceptance that others might be in similar situations. Link workers offered the ‘opportunities for activities, which allowed people to meet and socialise in the community’,^21^ providing an initial introduction to others. All six studies reported how service users felt social prescribing services had allowed them to develop new friendships, establish group identities, and reconnect with old friends.^7,21–23,28,36^ The development of these relationships was reported to have led to positive changes in service users’ mental health management and wellbeing.

## Discussion

This systematic review synthesised six UK-based qualitative studies, all of which used thematic analysis of semi-structured interview data to capture service users’ experiences of social prescribing interventions.^7,21–23,28,36^

The importance of a person-centred care approach underpinned delivery of social prescribing. Themes were derived from the lived experience of service users encompassing personalised goal setting and tailoring of services to account for fluctuations in their mental health. Themes also covered the development of a therapeutic alliance, and referrals to services for activities that matched their mental health needs and preferences, including provision of a social and supportive environment. These components of social prescribing services all align closely with the principles of person-centred care.^38^ Research consistently reports that care matched to a person’s preferences and values leads to better engagement, adherence and satisfaction with treatment and services,^39,40^ while also promoting self-determination, choice and autonomy, which are core components of recovery-orientated practice.^41,42^ Principles of shared decision-making include a positive therapeutic alliance, which is a strong predictor of engagement in therapy^43^ and outcomes in case management services in community mental health.^44^

The development of supportive social environments, created by social prescribing services, allowed service users to build their own community and support network. This linked directly to the second analytical theme identified in this study, whereby service users described social prescribing as producing an environment conducive to supporting personal change and development by addressing their holistic health needs and improving their self-confidence and social interactions. A social environment aimed at reducing loneliness and increasing a sense of social connectiveness has been shown to have a positive impact on mental health,^26,45^ with greater numbers of group connections positively impacting on quality of life.^46^ Creating supportive environments for service users helps to build a sense of community, which can act as vital sources of peer support during fluctuations in mental health.^47^ Formation of friendships, as identified by all studies in this review, also arise through activities such as art or music, which in turn can positively impact on mental health.^46,47^

### Strengths and limitations

The application of thematic synthesis to review the evidence within the field of social prescribing represents a novel approach. This review also synthesised the views and experiences of service users across multiple studies, with a specific focus on how social prescribing supports adults experiencing difficulties with their mental health. It adds an analytical approach to understanding the essential components of social prescribing services from a service user viewpoint which has not been done before as part of a synthesis. Despite conducting a comprehensive search of the literature, one limitation of this review is the lack of a universal definition of ‘social prescribing’ and related medical subject headings in bibliographic databases. Therefore, the existence of studies that would have met our eligibility criteria cannot be ruled out. In addition, the nature of thematic synthesis is dependent on quality of reporting in published manuscripts. Analytical and descriptive themes reported in this review are created from data reported within the published version of the manuscript and other unpublished data of relevance may be available. Finally, five out of the six studies collected data from service users after they had engaged with social prescribing services. Therefore, our findings are less reflective of service user views during engagement in social prescribing services, including those accessing services that do not utilise link workers.

### Future research

It is vital for the sustainability of social prescribing services to be driven by service user experiences to maximise engagement in activities, and outcomes that matter to service users, including cost-effectiveness. However, few services explicitly report on involving service users in co-design/production.^13^ Future research would also benefit from assessing how different delivery styles/modes of delivery (i.e. over the phone, in-person, video call or a blended engagement approach) influences people’s experiences of person-centred delivery and outcomes. The perspective of link workers and referrers involved in social prescribing would also benefit from research to inform training and supervision. For example, to understand the skills employed by link workers and others that fosters a person-centred care delivery and environment. Link workers have described the complexity involved in their role (changing conditions, different levels of support required), and need to have regular supervision and/or engage in self-care practices to mitigate any negative impact on their well-being.^48,49^

## Conclusions

This application of thematic synthesis has provided a novel approach to the synthesis of qualitative evidence for service users’ experiences of social prescribing services to support their mental health. Adherence to principles of person-centred care and addressing holistic needs of service users, including devoting attention to the quality of the therapeutic environment, are important for the design and delivery social prescribing services to optimise service user satisfaction and other outcomes that matter to them.

## Supplemental Material

sj-docx-1-rsh-10.1177_17579139231170786 – Supplemental material for Service user perspectives on social prescribing services for mental health in the UK: a systematic review Click here for additional data file. Supplemental material, sj-docx-1-rsh-10.1177_17579139231170786 for Service user perspectives on social prescribing services for mental health in the UK: a systematic review by M Cooper, D Flynn, L Avery, K Ashley, C Jordan, L Errington and J Scott in Perspectives in Public Health

sj-docx-2-rsh-10.1177_17579139231170786 – Supplemental material for Service user perspectives on social prescribing services for mental health in the UK: a systematic review Click here for additional data file. Supplemental material, sj-docx-2-rsh-10.1177_17579139231170786 for Service user perspectives on social prescribing services for mental health in the UK: a systematic review by M Cooper, D Flynn, L Avery, K Ashley, C Jordan, L Errington and J Scott in Perspectives in Public Health

sj-docx-3-rsh-10.1177_17579139231170786 – Supplemental material for Service user perspectives on social prescribing services for mental health in the UK: a systematic review Click here for additional data file. Supplemental material, sj-docx-3-rsh-10.1177_17579139231170786 for Service user perspectives on social prescribing services for mental health in the UK: a systematic review by M Cooper, D Flynn, L Avery, K Ashley, C Jordan, L Errington and J Scott in Perspectives in Public Health

sj-docx-4-rsh-10.1177_17579139231170786 – Supplemental material for Service user perspectives on social prescribing services for mental health in the UK: a systematic review Click here for additional data file. Supplemental material, sj-docx-4-rsh-10.1177_17579139231170786 for Service user perspectives on social prescribing services for mental health in the UK: a systematic review by M Cooper, D Flynn, L Avery, K Ashley, C Jordan, L Errington and J Scott in Perspectives in Public Health
