# Supplementary material for: Service user perspectives on social prescribing services for mental health in the UK: a systematic review
Source: Perspect Public Health. 2023 May 26;143(3):135–44. doi: 10.1177/17579139231170786 (PMC10226005; doi:10.1177/17579139231170786)
Supplement: sj-docx-2-rsh-10.1177_17579139231170786 – Supplemental material for Service user perspectives on social prescribing services for mental health in the UK: a systematic review [file sj-docx-2-rsh-10.1177_17579139231170786.docx]

**Supplementary Material 2**

**Search strategy used**

**Cochrane search:**

("mental health" OR "mental disease*" OR "mental disorder*" OR anxiety OR bipolar OR "disruptive impulse control" OR "conduct disorder*" OR "dissociative disorder*" OR "eating disorder*" OR "feeding disorder*" OR "mood disorder*" OR "personality disorder*" OR "somatoform disorder*" OR trauma OR "stress* related disorder*" OR depression OR wellbeing OR well-being OR "psychiatric disorder*" OR "psychiatric problem" OR "non-medical symptom*" OR "psychosocial problem" OR "psycho-social problem" OR mups OR "medically unexplained physical symptom*" OR "mental difficult*" OR recovery OR "social function*"):ti,ab,kw

AND

## (social near/4 (prescri* OR referral OR intervention)):ti,ab,kw OR (community near/4 (prescri* OR referral OR intervention)):ti,ab,kw OR ("linking scheme*" OR u3a OR "university of the third age" OR "buddy scheme*" OR "men's shed" OR ecotherapy OR "individual placement" OR "supported employment" OR "non-medical referral" OR "non-clinical referral"):ti,ab,kw OR ((wellbeing near/2 referral)):ti,ab,kw OR ((well-being near/2 referral)):ti,ab,kw

**Scopus Search:**

( ( ( TITLE-ABS-KEY ( "mental health" OR "mental disease*" OR "mental disorder*" OR anxiety OR bipolar OR "disruptive impulse control" OR "conduct disorder*" OR "dissociative disorder*" OR "eating disorder*" OR "feeding disorder*" OR "mood disorder*" OR "personality disorder*" ) OR TITLE-ABS-KEY ( "somatoform disorder*" OR trauma OR "stress* related disorder*" OR "mental* ill*" OR depression OR wellbeing OR well-being OR "psychiatric disorder*" OR "psychiatric problem" ) ) ) OR ( TITLE-ABS-KEY ( "non-medical symptoms" OR psychosocial OR psycho-social OR mups OR "medically unexplained physical" OR "mental difficult*" OR recovery OR "social function*" ) ) ) AND ( ( ( TITLE-ABS-KEY ( social W/4 ( prescri* OR referral OR intervention ) ) ) OR ( TITLE-ABS-KEY ( community W/4 ( prescri* OR referral OR intervention ) ) ) OR ( TITLE-ABS-KEY ( "linking scheme*" OR u3a OR "university of the third age" OR "buddy scheme*" OR "men's shed" ) ) ) OR ( TITLE-ABS-KEY ( ecotherapy OR "individual placement" OR "supported employment" OR "non-medical referral" OR "non-clinical referral" ) ) )

**Web of Science Search:**

( ( TS=( prescri* near/4 ( exercis* OR education OR learning OR arts ) ) ) OR ( TS=( "information referral" OR "social referral" OR "green gym" OR "sign-posting intervention" OR "healthy living" OR "time bank" OR "supported referral" OR "non-clinical intervention" OR ecotherapy OR "employment skills" OR "individual placement" ) ) OR ( TS=( "supported employment" OR "non-medical referral" OR "non-clinical referral" ) ) OR ( TS=( wellbeing near/2 referral ) ) OR (TS=( well-being near/2 referral ) ) OR ( ( TS=( social near/4 ( prescri* OR referral OR intervention) ) ) OR ( TS=( community near/4 ( prescri* OR referral OR intervention) ) ) OR ( TS=( "linking scheme*" OR u3a OR "university of the third age" OR "buddy scheme*" OR "men's shed") ) ) ) AND ( ( ( TS=( "mental health" OR "mental disease*" OR "mental disorder*" OR anxiety OR bipolar OR "disruptive impulse control" OR "conduct disorder*" OR "dissociative disorder*" OR "eating disorder*" OR "feeding disorder*" OR "mood disorder*" OR "personality disorder*" ) OR TS=( "somatoform disorder*" OR trauma OR "stress* related disorder*" OR "mental* ill*" OR depression OR wellbeing OR well-being OR "psychiatric disorder*" OR "psychiatric problem") ) ) OR ( TS=( "non-medical symptoms" OR "psychosocial problem" OR "psycho-social problem" OR mups OR "medically unexplained physical" OR "mental difficult*" OR "ill health" OR recovery OR "social function*" ) ) )

Indexes=SCI-EXPANDED, SSCI, A&HCI, CPCI-S, CPCI-SSH, ESCI Timespan=All years

**Medline/ Embase/ PsychINFO search:**

1 (Social adj4 (prescri* or referral or intervention)).mp.

2 (community adj4 (prescri* or referral or intervention)).mp.

3 linking scheme*.mp.

4 u3a.mp.

5 university of the third age.mp.

6 buddy scheme*.mp.

7 men's shed.mp.

8 (prescri* adj4 (exercis* or education or learning or arts)).mp.

9 information referral.mp.

10 social referral.mp.

11 green gym.mp.

12 time bank.mp.

13 supported referral.mp.

14 (well-being adj2 referral).mp.

15 (wellbeing adj2 referral).mp.

16 ecotherapy.mp.

17 Individual Placement.mp.

18 supported employment.mp.

19 non-medical referral.mp.

20 non-clinical referral.mp.

21 or/1-20

22 Mental Health/

23 mental disorders/ or anxiety disorders/ or "bipolar and related disorders"/ or "disruptive, impulse control, and conduct disorders"/ or dissociative disorders/ or "feeding and eating disorders"/ or mood disorders/ or personality disorders/ or somatoform disorders/ or "trauma and stressor related disorders"/

24 mental* ill*.mp.

25 Depression/

26 exp Anxiety/

27 wellbeing.mp.

28 well-being.mp.

29 psychiatric disorder*.mp.

30 psychiatric problem.mp.

31 non-medical symptoms.mp.

32 psycho-social problem*.mp.

33 psychosocial problem*.mp.

34 mups.mp.

35 medically unexplained physical symptoms.mp.

36 non-medical problem.mp.

37 mental difficult*.mp.

38 recovery.mp.

39 Mental Health Recovery/

40 social function*.mp.

41 or/22-40

42 21 and 41

***************************
