# Supplementary material for: Service user perspectives on social prescribing services for mental health in the UK: a systematic review
Source: Perspect Public Health. 2023 May 26;143(3):135–44. doi: 10.1177/17579139231170786 (PMC10226005; doi:10.1177/17579139231170786)
Supplement: sj-docx-3-rsh-10.1177_17579139231170786 – Supplemental material for Service user perspectives on social prescribing services for mental health in the UK: a systematic review [file sj-docx-3-rsh-10.1177_17579139231170786.docx]

**Supplementary materials 3**

Methodological Quality Assessment

|  | **Hanlon et al. 2021 [37]** | **Kellezi et al. 2019 [7]** | **Moffatt et al. 2017 [21]** | **Roberts et al, 2020 [29]** | **Stickley et al. 2012 [24]** | **Wildman et al. 2019 [22]** |
| --- | --- | --- | --- | --- | --- | --- |
| **Was there a clear statement of the aims of the research?** | Yes (2 points) | Yes (2 points) | Yes (2 points) | Yes (2 points) | Yes (2 points) | Yes (2 points) |
| **Is a qualitative methodology appropriate?** | Yes (2 points) | Yes (2 points) | Yes (2 points) | Yes (2 points) | Yes (2 points) | Yes (2 points) |
| **Was the research design appropriate to address the aims of the research?** | Yes (2 points) | Yes (2 points) | Yes (2 points) | Yes (2 points) | Yes (2 points) | Yes (2 points) |
| **Was the recruitment strategy appropriate to the aims of the research?** | Yes (2 points) | Yes (2 points) | Yes (2 points) | Yes (2 points) | Yes (2 points) | Yes (2 points) |
| **Was the data collected in a way that addressed the research issue?** | Yes (2 points) | Yes (2 points) | Yes (2 points) | Yes (2 points) | Yes (2 points) | Yes (2 points) |
| **Has the relationship between researcher and participants been adequately considered?** | Yes (2 points) | No (0 points) | No (0 points) | No (0 points) | Can’t Tell (1 point) | No (0 points) |
| **Have ethical issues been taken into consideration?** | Yes (2 points) | Can’t Tell –  no ethical statement (1 point) | Yes (2 points) | Can’t Tell (1 point) | Yes (2 points) | Yes (2 points) |
| **Was the data analysis sufficiently rigorous?** | Yes (2 points) | Yes (2 points) | Yes (2 points) | Can’t Tell (1 point) | Yes (2 points) | Yes (2 points) |
| **Is there a clear statement of findings?** | Yes (2 points) | Yes (2 points) | Yes (2 points) | Yes (2 points) | Yes (2 points) | Yes (2 points) |
| **How Valuable is the research** | Discussed contribution to knowledge – YES,  identify new areas- YES,  Transferability -YES  Conclusion- Very Valuable (2 points) | Discussed contribution to knowledge – YES,  identify new areas- YES,  Transferability -YES  Conclusion- Very Valuable (2 points) | Discussed contribution to knowledge – YES,  identify new areas- YES,  Transferability -YES  Conclusion- Very Valuable (2 points) | Discussed contribution to knowledge – YES,  identify new areas- Can’t Tell,  Transferability -YES  Conclusion-  Valuable  (1 point) | Discussed contribution to knowledge – YES,  identify new areas- YES  Transferability -YES  Conclusion-  Very Valuable (2 points) | Discussed contribution to knowledge – YES,  identify new areas- YES  Transferability -YES  Conclusion-  Very Valuable (2 points) |
| **Total Score** | **20** | **17** | **18** | **15** | **19** | **18** |
