# Supplementary material for: Service user perspectives on social prescribing services for mental health in the UK: a systematic review
Source: Perspect Public Health. 2023 May 26;143(3):135–44. doi: 10.1177/17579139231170786 (PMC10226005; doi:10.1177/17579139231170786)
Supplement: sj-docx-4-rsh-10.1177_17579139231170786 – Supplemental material for Service user perspectives on social prescribing services for mental health in the UK: a systematic review [file sj-docx-4-rsh-10.1177_17579139231170786.docx]

| **Analytical Theme** | **Descriptive theme** | **Example Direct Codes** | **Example supporting quotes from results section of included papers [where available]** |
| --- | --- | --- | --- |
| **1.Person-centred approach was key to delivery** | **1.1 Developing therapeutic relationships with link workers was essential** | ‘An important aspect of the support patients received was having someone listen to them…an important aspect of the therapeutic relationship (which was echoed across accounts) when she notes that the link worker ‘wasn’t demanding’.Page5[7] | *“I felt as though they gave me the chance to reason out that I was getting better. I listened to them. I knew what was going on in my head, but I couldn’t always, I didn’t always want to tell anyone. I seemed, with the link- worker, I seemed as though I could get over that more quickly..”* |
|  |  | ‘Participants consistently reported feeling at ease and relaxed with their Link Worker, which enabled them to develop an open and trusting relationship’ Page 6[21] | *“we just started talking and that’s when I just came out with different things ... and she gave the feeling that you could open up to her”* |
|  |  | ‘For all participants in this study, the link worker was a central figure in their experience of social prescribing and the majority of participants had developed strong relationships with their link worker ‘Page 3[22] | *“I look at him [link worker] as like a pal. It’s as simple as that.”* |
|  |  | ‘Participants found a place of sanctuary and escape. The fact that they were not pressurized into participation meant that there was little pressure and this was greatly appreciated’ Page576[24] | *“We all felt as if we understood one another. So it worked beautifully.”* |
|  |  | ‘For many participants, what made the programme work was that the volunteer [link worker] was well-matched to the client. The kindness, patience, support and guidance of the volunteers were reassuring and helped increase confidence’ Page158[29] | *“The key things that make it work is the excellent volunteers and their patience with us. It is also good that they have a goal at the end of the ten weeks and the fact that they can refer people on to others”* |
|  |  | ‘For all participants describing improvements following the Links Worker Programme the relationship with the [link worker] was central to their narrative’ Page 179[37] | *“when you’re actually going tae the doctor because you’re no’ functioning properly day-tae-day, an’ somebody takes a’ the bits that you struggle with, they’re a lifesaver. It’s better than any betablocker.”* |
|  | **1.2 Link workers should ensure onward referrals are appropriate and person centred** | ‘Typically, participants did not feel positive about the [social prescribing] pathway when they felt the groups they were referred to did not meet their needs or they felt unwelcomed’ Page 6[7] | *“Whoever’s running a particular class should be made aware of the programme itself and the issues and the impact it could have on the people who have eventually managed to get out of the house, and treat them a little better”* |
|  |  | ‘Long-term condition management services that service users were directed to by Link Workers were highlighted as extremely helpful, particularly the combination of expert and peer-led advice on coping and symptom management strategies’ Page 8[21] | *“that has been absolutely wonderful ... honestly it was the turning point in my life. Because I had reached rock bottom and I just needed practical help to try and get back up ... you have to manage your expectations as well as your illness and it has helped considerably.”* |
|  |  | ‘The appropriateness of onward referrals to voluntary and community groups was also important for continued engagement with the intervention’ Page 3[22] | *“The recipes she gave us were the type of food we wouldn’t have eaten anyway. We’ve realised we can’t change our food. We can’t. I’ve tried. I will lose weight if I eat meals like an English person eats.”* |
|  |  | ‘By feeling accepted and amongst people with similar experiences participants gained a sense of social belonging, which engendered spontaneous peer support and helped establish a sense of group identity and ultimately friendship.’ Page 577[24] | *“They all come together and everybody does their little bit that is what this represents e bringing people together, sharing under- standing and sharing it within the community. And it’s price- less.”* |
|  |  | ‘In addition to the quality of the volunteers [link workers], the fact that Cadwyn Môn refers clients on to other services and groups during the programme was noted as something that adds to its effectiveness.’ Page 158[29] | *“The key things that make it work is the excellent volunteers and their patience with us. It is also good that they have a goal at the end of the ten weeks and the fact that they can refer people on to others such as the Red Cross or WRVS”* |
|  |  | ‘These relationships were often formed through group activities which had been suggested or organized by the [link workers]. These wider relationships tended to positively impact wellbeing ‘Page 180[37] | *“mixing wi’ with people was difficult for me. . . people [were] talking to you and they were suffering fae anxiety and that as well. . . It made me feel like, I’m not the only person out there... it is helping me. My anxiety’s a lot better.”* |
|  | **1.3 Personalised goal setting supported progress** | ‘Participants saw this [link worker approach] as a goal achievement facilitator. When discussing the progress of their goals with staff, beneficiaries experienced support as encouraging rather than punitive [contrary to their expectations].’ Page5[7] | *“He wasn’t demanding. He was very quiet and very gentle with it, and that is the way that I needed somebody to be, to maybe listen to me, really listen to me, and hear what I was saying, if you can understand that”* |
|  |  | ‘It supported realistic, progressive and personalised goal-setting. Participants’ expectations of progress were therefore achievable and reflected that a long-term approach was necessary to make improvements, helping people to live with their conditions and improve their well-being’ Page 8[21] | *“they have a very practical approach and know that it has got to be incremental ... you can’t do everything at once so you have got to start small and build up ... they have got the big picture in mind but lots of little steps in between to get there”* |
|  |  | ‘The emphasis on gradual change was identified as particularly valuable, enabling the setting of “mini-goals” that represented “achievable somethings” in order to make progress towards goals such as a return to employment’ Page 7[22] | No further in text quote[s] provided |
|  |  | ‘Many described how the [Community Links Practitioner] allowed them to voice their priorities and have control over what goals were set. This was seen as a contrast to many of the other interactions they experienced other areas of their lives’ Page 182[37] | *“naebody’s telling you you’ve got to do this, you’ve got to do that.”* |
|  | **1.4 Tailoring of services could mitigate impact of health fluctuations on engagement** | ‘Regularly engaging with services was challenging, particularly for people whose condition fluctuated and those suffering from more than one health problem. Service users worried about not always being able to attend, which was often a reason for not sustaining engagement with services in the past’ Page 8[21] | *“I was really down, so I couldn't go on Friday. It's been like that since I first joined. I've been missing [sessions] because of my COPD ... bad turns ... chest infections ... [but] they [Ways to Wellness] restarted me again, so they've looked after me ... they've been really, really good”* |
|  |  | ‘Unanticipated health shocks or trauma could impact on progress and this could be demoralising…also reported was the psychological burden of living with LTCs, which could create barriers to progress that could be as strong, or stronger, than the physical impact of a condition. For participants with depression and anxiety, motivation to try new groups and activities could be a particular problem’ Page 8[22] | *“As I say, I just haven’t got the energy, not at all. You get the days where you are feeling dead down and you can’t be bothered. That is the way I feel at the minute...she [link worker] has asked me to join groups and different things. There are things I want to do, but over these last few months I just haven’t had the energy, I just didn’t want to go or even mix with people.”* |
| **2. Creating an environment for personal change and development** | **2.1 Social prescribing provided a holistic view of health and support** | ‘they believed that social prescribing was qualitatively different from their experiences with other health professionals’ Page 5[7] | *“I think when you go to the doctor, you're used to having this ten-minute slot and you have to like quickly get everything in. And then when you go and see a counsellor, or you go and see your support worker, you have that full hour, and I wasn't really used to that at the time, that expanse of time where you can just relax and talk.”* |
|  |  | ‘The holistic service offered by the link workers was contrasted favourably with what was available or possible through the GP or with previous attempts by other health professionals to deal with complex health issues’ Page 7[21] | *“At the very beginning, we agreed that she’d work around me, not me around her, to work on the foods that I thought were suitable for me ... I met up with dieticians before ... I felt they were dictating the food I should have, not the food I wanted to have. So that's what surprised me about Ways to Wellness, they're working around me ... it’s different. You talk about it. You're relaxed ... You're in charge of it”* |
|  |  | ‘As adults living with LTCs in an area of socioeconomic deprivation, many participants had problems beyond LTC management alone and reported receiving support from their link worker across a number of areas, including housing, debt and welfare benefits’ Page 6[22] | *“They've helped me, sorted my finances and that out and they helped me with getting in touch with certain groups of people on my finances, which I was worried about at the time. That’s getting sorted. That got sorted. They helped me in a lot of different ways because I thought I was losing my mind and that, but I think I'm getting a bit better.”* |
|  |  | ‘For some individuals the opportunity for personal development brought hope and meaning to life. For others, engagement with Arts on Prescription provided a distraction from a variety of problems in life’ Page 577[24] | *“You don’t have to think about anything else and how hard life is; relaxation makes it sound lazy but it is very calming and relaxing and that has to be a good thing”* |
|  |  | ‘Participants said they were more confident, happier and feeling better with an improved outlook on life since receiving the service’ Page158[29] | *“I have changed in the last ten weeks, I am much happier having found some places to go”* |
|  | **2.2 Service users were able to develop their self-confidence and social interactions** | ‘Many patients described increased feelings of self- confidence following their pathway participation. This was particularly evident for those with complex conditions and/or social isolation.’ Page 6[7] | No further in text quote[s] provided |
|  |  | ‘Building self-confidence, self-reliance and independence was another facet of the Link Workers’ approach, managed through ongoing support and persistence in finding the right motivational tools for the individual, while conveying the need for personal responsibility and resilience. This enabled service users to make changes to their lives, engage with other organisations and manage their long-term conditions’ Page 7[21] | *'I mean, she’s just someone who can make you do things… Not as in a bad way, she sort of, like, empowers you, for want of a better word, to do it, you know what I mean? She gives you, ‘… give it a go,’ and she’ll explain… and if you don’t go she doesn’t get disappointed or anything like that, she just says, ‘Oh, right, well, we’ll sort something else out for you.”… I think the only person that can help is myself, really. I think it’s 99% me and the 1% of help I get from the rest of the people, but … they can only like, give you advice and encouragement, the rest has got to be yourself.'* |
|  |  | ‘participants noted that their confidence and ability to self-manage LTCs had improved. Many participants felt confident they could continue with the coping strategies and changes they had made earlier in their engagement with the intervention, or at least were growing in confidence’ Page 6[22] | No further in text quote[s] provided |
|  |  | ‘People had the opportunity to re-build and re-establish themselves, and to re-evaluate past negative experiences. New skills were developed and these skills created confidence, both socially and artistically.’ Page 577[24] | *“If you can create something, it gives you that sense of purpose, a bit more self confidence that you’re not going to get elsewhere”* |
|  |  | ‘These results suggest that overall, from the perspective of those who took part, the Cadwyn Môn programme was successful in increasing confidence and facilitated social connectedness in the community.’ Page158[29] | No further in text quote[s] provided |
|  |  | ‘Patients who rediscovered a sense of competence in an activity often described an increased sense of confidence and control over other areas of life’ Page 180[37] | *“I never went oot anywhere and then after she died, I didnae go oot ‘cause I’d lost all my conﬁdence and everything...I can walk in and out of that room now, so I can, without it feeling bad or anything like that. And I think it is all doon to this group thing.”* |
|  | **2.3 Service users benefited from peer support** | ‘A positive group experience was also vital. This was typically facilitated by a sense of belonging and feeling welcomed by the group (and leader). Aside from loneliness alleviation, groups allowed participant 4 to provide support to similar others, which he experienced as an important aspect of group membership’ Page 6[7] | *“You’re kind of helping each other, because I think for most people [with this condition] you kind of feel that you’re the only person on the whole of Plant Earth, you know. You don’t seem to know how many other people [have this condition] so the fact that you can meet up with others is like, oh, there are other people that understand and know how it’s difficult (…) and so, you were able to give each other encouragement or copy each other or learn from each other.”* |
|  |  | ‘Offering opportunities for activities, which allowed people to meet and socialise in the community, reduced social isolation and positively impacted on self-confidence, self-esteem and mental well-being’ Page 7[21] | *“After [partner] passed away I was, not a recluse, but I just didn’t want to talk to anybody. But since I’ve been coming to see [the Link Worker] I’ve broadened my horizons and I get out … I’ve got a lot more confidence”* |
|  |  | ‘For socially isolated participants, increased social contact and the chance to make friends with people in a similar situation was a motivating factor for continued involvement’ Page 7[22] | *“So you get to know people every week you're there [community group]…I have to come or they will keep saying, “Are you coming next week? Are you doing this? Are you involved?” So other people are asking me will I be there. The friends that I've taken or whatever.”* |
|  |  | ‘By feeling accepted and amongst people with similar experiences participants gained a sense of social belonging, which engendered spontaneous peer support and helped establish a sense of group identity and ultimately friendship’ Page 557[24] | *“They all come together and everybody does their little bit that is what this represents e bringing people together, sharing understanding and sharing it within the community. And it’s priceless.”* |
|  |  | ‘re-connecting with old friends and making new ones were prominent themes. Participants reported making new friends in the classes and groups that they had joined with the assistance of Cadwyn Môn, and others had been able to get in touch and meet up with old friends’ Page 158[29] | *“My social life has become busy and I have made new friends”* |
|  |  | ‘All patients with moderate or major improvement developed positive relationships with other people following their interaction with the CLP. This was described as a change from the isolation experienced prior to the intervention…These  wider relationships tended to positively impact wellbeing’ Page 180[37] | *“mixing wi’ with people was difﬁcult for me.. . people [were] talking to you and they were suffering fae anxiety and that as well.. . It made me feel like, I’m not the only person out there... it is helping me. My anxiety’s a lot better.”* |
